# Supplementary material for: An open-source framework for large-scale, flexible evaluation of biomedical text mining systems
Source: J Biomed Discov Collab. 2008 Jan 29;3:1. doi: 10.1186/1747-5333-3-1 (PMC2276192; doi:10.1186/1747-5333-3-1)
Supplement: Additional file 1 — Evaluation platform source code. The additional file contains the software evaluation framework discussed in this paper. The most current release can be downloaded from SourceForge.net [1]. See the accompanying README file for instructions on installation and use. Also included in the distribution is a collection of UIMA wrappers for some commonly used BioNLP tools and annotated corpora. [file 1747-5333-3-1-S1.zip › bionlp_uima_repository-1.0.1/docs/api/src-html/edu/uchsc/ccp/util/nlp/parser/corpus/YapexCorpusParser.html]

```
001    package edu.uchsc.ccp.util.nlp.parser.corpus;
002    
003    import java.io.BufferedReader;
004    import java.io.IOException;
005    import java.util.ArrayList;
006    import java.util.Iterator;
007    import java.util.List;
008    import java.util.regex.Matcher;
009    import java.util.regex.Pattern;
010    
011    import org.jdom.Document;
012    import org.jdom.Element;
013    import org.jdom.JDOMException;
014    import org.jdom.input.SAXBuilder;
015    
016    import edu.uchsc.ccp.util.nlp.GenericTextUtils;
017    import edu.uchsc.ccp.util.nlp.annotation.AnnotationSet;
018    import edu.uchsc.ccp.util.nlp.annotation.Annotator;
019    import edu.uchsc.ccp.util.nlp.annotation.TextAnnotation;
020    import edu.uchsc.ccp.util.nlp.document.GenericDocument;
021    import edu.uchsc.ccp.util.nlp.ei.xml.XMLEntityExtractor;
022    import edu.uchsc.ccp.util.nlp.mention.ClassMentionTypes;
023    import edu.uchsc.ccp.util.xml.XMLValidityChecker;
024    
025    /**
026     * This class is used to parse out the gold standard annotations from the Yapex corpus.<br>
027     * <br>
028     * Output Classes:<br>
029     * <ul>
030     * <li>protein</li>
031     * </ul>
032     * 
033     * @author williamb
034     * 
035     */
036    
037    public class YapexCorpusParser extends CorpusParser {
038        public static final int DOCUMENT_COLLECTION_ID = 7;
039        
040        private final String PUBMED_ARTICLE_SET_TAG = "PubmedArticleSet";
041    
042        private final String PUBMED_ARTICLE_TAG = "PubmedArticle";
043    
044        private final String MEDLINE_ID_TAG = "MedlineID";
045    
046        private final String PMID_TAG = "PMID";
047    
048        private final String ARTICLE_TITLE_TAG = "ArticleTitle";
049    
050        private final String ABSTRACT_TEXT_TAG = "AbstractText";
051    
052        private final String PROTEIN_TAG = "Protname";
053    
054        private boolean isTest = false;
055    
056        private BufferedReader corpusReader;
057    
058        private Annotator annotator;
059    
060        private AnnotationSet annotationSet;
061    
062        private List<String> extractedProteinNames;
063    
064        private List<TextAnnotation> allYapexAnnotations;
065    
066    
067    
068        private String connectionPropertiesFile;
069    
070        private static int annotationID = 0;
071    
072        private GenericDocument nextTitleDocument = null;
073    
074        private GenericDocument nextAbstractDocument = null;
075    
076        private boolean returnedTitleDocument = false;
077    
078        private boolean returnedAbstractDocument = false;
079    
080        private Iterator childrenIterator;
081    
082        /* String[] is of size 2. String[0] = docID (-t for title, -a for abstract) String[1] = text */
083        private List<String[]> allYapexDocuments;
084    
085    //    /**
086    //     * The constructor to use to parse the corpus and load the results in a database
087    //     * 
088    //     * @param corpusFile
089    //     * @param isTest
090    //     * @param connectionPropertiesFile
091    //     * @throws IOException
092    //     */
093    //    public YapexCorpusParser(String corpusFile, boolean isTest, String connectionPropertiesFile) throws IOException {
094    //
095    //        /* Is this the training set or the test set? */
096    //        this.isTest = isTest;
097    //        this.connectionPropertiesFile = connectionPropertiesFile;
098    //
099    //        /* initialize corpus reader for extracting tag offsets */
100    //        corpusReader = GenericTextUtils.openReader(corpusFile);
101    //
102    //        /* initialize annotator */
103    //        annotator = new Annotator(new Integer(9), "", "Yapex", "SICS");
104    //
105    //        /* initialize annotationset */
106    //        if (isTest) {
107    //            annotationSet = new AnnotationSet(new Integer(6), "Yapex test set annotations",
108    //                    "Annotations extracted from the test set documents of the Yapex corpus.");
109    //        } else {
110    //            annotationSet = new AnnotationSet(new Integer(5), "Yapex training set annotations",
111    //                    "Annotations extracted from the training set documents of the Yapex corpus.");
112    //        }
113    //
114    //        /* initialize storage for output annotations and document text - to be put into the database eventually */
115    //        allYapexAnnotations = new ArrayList<TextAnnotation>();
116    //        allYapexDocuments = new ArrayList<String[]>();
117    //
118    //        /* parse the corpus file, creating annotations as you go */
119    //        try {
120    //            parseCorpusFile(corpusFile);
121    //        } catch (IOException e) {
122    //            System.err.println("ERROR -- YapexCorpusParser: IOException while parsing corpus file.");
123    //            e.printStackTrace();
124    //        } catch (JDOMException e) {
125    //            System.err.println("ERROR -- YapexCorpusParser: JDOMException while parsing corpus file.");
126    //            e.printStackTrace();
127    //        }
128    //    }
129    
130        /**
131         * The constructor for the iterator-like corpus parser.
132         * 
133         * @param corpusFile
134         */
135        public YapexCorpusParser(String corpusFile, boolean isTest) throws IOException {
136            /* initialize annotator */
137            annotator = new Annotator(new Integer(9), "", "Yapex", "SICS");
138    
139            /* initialize corpus reader for extracting tag offsets */
140            corpusReader = GenericTextUtils.openReader(corpusFile);
141    
142            /* initialize annotationset */
143            if (isTest) {
144                annotationSet = new AnnotationSet(new Integer(6), "Yapex test set annotations",
145                        "Annotations extracted from the test set documents of the Yapex corpus.");
146            } else {
147                annotationSet = new AnnotationSet(new Integer(5), "Yapex training set annotations",
148                        "Annotations extracted from the training set documents of the Yapex corpus.");
149            }
150    
151            /*
152             * initialize storage for output annotations and document text - to be put into the database eventually - these are only initialized here
153             * because the code we are using is also the code used to store stuff in the database. It should be separated at some point.
154             */
155            allYapexAnnotations = new ArrayList<TextAnnotation>();
156            allYapexDocuments = new ArrayList<String[]>();
157    
158            if (!XMLValidityChecker.validateXML(corpusFile)) {
159                error("Yapex corpus file is not valid XML. Exiting...");
160                System.exit(-1);
161            } else {
162                try {
163                    initialize(corpusFile);
164                } catch (JDOMException e) {
165                    error("JDOMException while parsing the Yapex corpus file. System exiting...");
166                    e.printStackTrace();
167                    System.exit(-1);
168                }
169            }
170        }
171    
172        @Override
173        public boolean hasNext() {
174            /* if we have not yet returned the abstract document, then return true */
175            if (returnedTitleDocument && !returnedAbstractDocument) {
176                return true;
177            } else {
178                /* we have already returned the abstract document, so check to see if there are new title and abstract documents to return */
179                try {
180                    return hasNextDocument();
181                } catch (IOException e) {
182                    error("Error while parsing the Bio1 corpus file.");
183                    e.printStackTrace();
184                    return false;
185                }
186            }
187        }
188    
189        @Override
190        public GenericDocument next() {
191            if (!returnedTitleDocument) {
192                returnedTitleDocument = true;
193                return nextTitleDocument;
194            } else {
195                returnedAbstractDocument = true;
196                return nextAbstractDocument;
197            }
198        }
199    
200        /**
201         * Return true if there is another document to return
202         * 
203         * @return
204         * @throws IOException
205         */
206        private boolean hasNextDocument() throws IOException {
207            returnedTitleDocument = false;
208            returnedAbstractDocument = false;
209    
210            while (childrenIterator.hasNext()) {
211                Element childElement = (Element) childrenIterator.next();
212                if (childElement.getName().equals(PUBMED_ARTICLE_TAG)) {
213                    processArticle(childElement);
214                    return true;
215                } else {
216                    System.err.println("WARNING -- YapexCorpusParser: Unexpected element encountered: " + childElement.getName());
217                }
218            }
219            return false;
220        }
221    
222        /**
223         * Initialize the XML Parser for parsing the yapex corpus document
224         * 
225         * @param corpusFile
226         * @throws IOException
227         * @throws JDOMException
228         */
229        private void initialize(String corpusFile) throws IOException, JDOMException {
230            SAXBuilder saxBuilder = new SAXBuilder();
231            Document document = saxBuilder.build(corpusFile);
232            Element rootElement = document.getRootElement();
233    
234            childrenIterator = rootElement.getChildren().iterator();
235        }
236    
237    //    private void parseCorpusFile(String corpusFile) throws IOException, JDOMException {
238    //        /* check for valid xml */
239    //        if (XMLValidityChecker.validateXML(corpusFile)) {
240    //
241    //            SAXBuilder saxBuilder = new SAXBuilder();
242    //            Document document = saxBuilder.build(corpusFile);
243    //            Element rootElement = document.getRootElement();
244    //
245    //            Iterator childrenIterator = rootElement.getChildren().iterator();
246    //
247    //            while (childrenIterator.hasNext()) {
248    //                Element childElement = (Element) childrenIterator.next();
249    //                if (childElement.getName().equals(PUBMED_ARTICLE_TAG)) {
250    //                    processArticle(childElement);
251    //                } else {
252    //                    System.err.println("WARNING -- YapexCorpusParser: Unexpected element encountered: " + childElement.getName());
253    //                }
254    //            }
255    //
256    //            /* now add documents to the database */
257    //            for (String[] idTextPair : allYapexDocuments) {
258    //                String documentID = idTextPair[0];
259    //                String documentText = idTextPair[1];
260    //
261    //                documentText = documentText.replaceAll("'", "''");
262    //
263    //                char TorA = documentID.charAt(documentID.length() - 1);
264    //                String testOrTrain = "";
265    //                if (isTest) {
266    //                    testOrTrain = "test";
267    //                } else {
268    //                    testOrTrain = "train";
269    //                }
270    //
271    //                String sqlStr = "INSERT INTO YAPEX_CORPUS VALUES ('" + documentID + "', '" + Character.toUpperCase(TorA) + "', '"
272    //                        + testOrTrain + "', '" + documentText + "')";
273    //                System.out.println("SQLSTR: " + sqlStr);
274    //                Database_Util.execute(connectionPropertiesFile, sqlStr);
275    //            }
276    //
277    //            String sqlldrFileName = "";
278    //            String runLoadFileName = "";
279    //            if (isTest) {
280    //                sqlldrFileName = "yapex_testAnnotations.sqlldr";
281    //                runLoadFileName = "loadYapexTestAnnotations.sh";
282    //            } else {
283    //                sqlldrFileName = "yapex_trainAnnotations.sqlldr";
284    //                runLoadFileName = "loadYapexTrainAnnotations.sh";
285    //            }
286    //            /* add the annotations to the database in the form of an sqlldr file */
287    //            /* create the sqlldr input file */
288    //            PrintStream sqlldrFileStream = GenericTextUtils.openPrintStream(AnnotationDBProperties.EXTERNAL_BASE_ANNOTATION_DIR
289    //                    + "/inputFiles/" + sqlldrFileName);
290    //            AnnotationDB_Util.printTextAnnotationListToSqlldrFile(sqlldrFileStream, allYapexAnnotations, new ArrayList<String>(), true,
291    //                    AnnotationDBProperties.CONNECTION_PROPERTIES_FILE, false);
292    //            sqlldrFileStream.close();
293    //
294    //            /* add the parsed gene annotations to the database */
295    //            AnnotationDB_Util.loadAnnotationsIntoDB(AnnotationDBProperties.EXTERNAL_BASE_ANNOTATION_DIR,
296    //                    AnnotationDBProperties.LOCAL_BASE_ANNOTATION_DIR, sqlldrFileName, AnnotationDBProperties.SQLLDR_CONTROL_FILE_TEMPLATE,
297    //                    runLoadFileName, AnnotationDBProperties.ANNOTATION_DB_STATUS_FLAG_FILE, true);
298    //
299    //        } else {
300    //            System.err.println("ERROR -- YapexCorpusParser: Corpus file is not well-formed XML. Please fix and try again.");
301    //        }
302    //    }
303    
304        private void processArticle(Element articleElement) throws IOException {
305    
306            GenericDocument gd = new GenericDocument();
307    
308            nextTitleDocument = new GenericDocument();
309            nextAbstractDocument = new GenericDocument();
310    
311            Iterator childrenIterator = articleElement.getChildren().iterator();
312    
313            while (childrenIterator.hasNext()) {
314                Element childElement = (Element) childrenIterator.next();
315    
316                if (childElement.getName().equals(PMID_TAG)) {
317                    String documentID = childElement.getValue();
318                    gd.setDocumentID(documentID);
319                    nextTitleDocument.setDocumentID(documentID + "-t");
320                    nextAbstractDocument.setDocumentID(documentID + "-a");
321                } else if (childElement.getName().equals(MEDLINE_ID_TAG)) {
322                    gd.addSecondaryDocumentID(childElement.getValue());
323                } else if (childElement.getName().equals(ARTICLE_TITLE_TAG)) {
324                    String titleText = childElement.getValue();
325                    gd.setTitleText(titleText);
326                    nextTitleDocument.setDocumentText(titleText);
327                    processArticleTitle(childElement);
328                } else if (childElement.getName().equals(ABSTRACT_TEXT_TAG)) {
329                    String abstractText = childElement.getValue();
330                    gd.setAbstractText(abstractText);
331                    nextAbstractDocument.setDocumentText(abstractText);
332                    processAbstractText(childElement);
333    
334                    List<TextAnnotation> extractedProteinAnnotations = getTagOffsets(gd.getDocumentID(), gd.getTitleText(), gd
335                            .getAbstractText());
336    
337                    /* validation extracted proteins */
338                    /* there should be an equal number of extracted protein annotations and names */
339                    if (extractedProteinAnnotations.size() != extractedProteinNames.size()) {
340                        System.err.println("ERROR -- YapexCorpusParser: Unequal number of extracted annotations and names: #annotations:"
341                                + extractedProteinAnnotations.size() + "  #names:" + extractedProteinNames.size() + " For PMID: "
342                                + gd.getDocumentID());
343                    }
344                    /* check to see that the annotation covered text matches the extracted name */
345                    for (int i = 0; i < extractedProteinAnnotations.size(); i++) {
346                        String coveredText = extractedProteinAnnotations.get(i).getCoveredText();
347                        String proteinName = extractedProteinNames.get(i);
348                        if (!coveredText.equals(proteinName)) {
349                            System.err.println("ERROR -- YapexCorpusParser: Covered text does not match extracted protein name.  CoveredText:"
350                                    + coveredText + " ProteinName:" + proteinName + "  PMID:" + gd.getDocumentID());
351                        }
352                    }
353    
354                    /* add the extracted annotations to the allYapexAnnotations list */
355                    allYapexAnnotations.addAll(extractedProteinAnnotations);
356                    gd.setAnnotations(extractedProteinAnnotations);
357    
358                    /* add the title and abstract text to the allYapexDocuments list */
359                    /* title */
360                    String[] t = new String[2];
361                    t[0] = gd.getDocumentID() + "-t";
362                    t[1] = gd.getTitleText();
363                    /* abstract */
364                    String[] a = new String[2];
365                    a[0] = gd.getDocumentID() + "-a";
366                    a[1] = gd.getAbstractText();
367    
368                    allYapexDocuments.add(t);
369                    allYapexDocuments.add(a);
370    
371                    // } else if (childElement.getName().equals(PROTEIN_TAG)) {
372    
373                } else {
374                    System.err.println("WARNING -- YapexCorpusParser: Unexpected element encountered: " + childElement.getName());
375                }
376            }
377    
378            // gd.printDocument(System.out);
379        }
380    
381        private void processArticleTitle(Element titleElement) {
382            /*
383             * we assume the title always comes before abstract - re-initialize the extractedProteinNames list. this will be used to check that we got
384             * everything from the getTagOffsets method
385             */
386            extractedProteinNames = new ArrayList<String>();
387            lookForProteinNames(titleElement);
388        }
389    
390        private void processAbstractText(Element abstractElement) {
391            lookForProteinNames(abstractElement);
392        }
393    
394        private void lookForProteinNames(Element textElement) {
395            Iterator childrenIterator = textElement.getChildren().iterator();
396    
397            while (childrenIterator.hasNext()) {
398                Element childElement = (Element) childrenIterator.next();
399                if (childElement.getName().equals(PROTEIN_TAG)) {
400                    String proteinName = childElement.getValue();
401                    extractedProteinNames.add(proteinName);
402                } else {
403                    System.err.println("WARNING -- YapexCorpusParser: Expected only protein tag. Unexpected element encountered: "
404                            + childElement.getName());
405                }
406            }
407    
408        }
409    
410        /* gets the offsets for the protein name tags for the next document */
411        private List<TextAnnotation> getTagOffsets(String referencePMID, String origTitleText, String origAbstractText) throws IOException {
412            String line;
413            String pmid = null;
414            String titleText = null;
415            String abstractText = null;
416            boolean foundTitle = false;
417            boolean foundAbstract = false;
418            boolean foundPMID = false;
419            while ((line = corpusReader.readLine()) != null & !line.equals("</PubmedArticle>")) {
420                if (line.startsWith("<PMID>")) {
421                    Pattern p = Pattern.compile("<PMID>(\\d+)</PMID>");
422                    Matcher m = p.matcher(line);
423                    if (m.find()) {
424                        pmid = m.group(1);
425                        foundPMID = true;
426                    } else {
427                        System.err.println("WARNING -- YapexCorpusParser: expecting pmid but did not match");
428                    }
429                } else if (line.startsWith("<ArticleTitle>")) {
430                    Pattern p = Pattern.compile("<ArticleTitle>(.*)</ArticleTitle>");
431                    Matcher m = p.matcher(line);
432                    if (m.find()) {
433                        titleText = m.group(1);
434                        foundTitle = true;
435                    } else {
436                        System.err.println("WARNING -- YapexCorpusParser: expecting title but did not match");
437                    }
438                } else if (line.startsWith("<AbstractText>")) {
439                    Pattern p = Pattern.compile("<AbstractText>(.*)</AbstractText>");
440                    Matcher m = p.matcher(line);
441                    if (m.find()) {
442                        abstractText = m.group(1);
443                        foundAbstract = true;
444                    } else {
445                        System.err.println("WARNING -- YapexCorpusParser: expecting abstract but did not match");
446                    }
447                }
448            }
449    
450            if (!foundPMID) {
451                System.err.println("WARNING -- YapexCorpusParser: did not find PMID");
452                return null;
453            }
454    
455            if (!foundTitle) {
456                System.err.println("WARNING -- YapexCorpusParser: did not find title");
457                return null;
458            }
459    
460            if (!foundAbstract) {
461                System.err.println("WARNING -- YapexCorpusParser: did not find abstract");
462                return null;
463            }
464    
465            /* make sure this is the correct document - the pmid found here must match the referencePMID input parameter */
466            if (pmid.equals(referencePMID)) {
467    
468                /* search for protein names in the title and abstract text */
469                List<TextAnnotation> titleAnnotations = XMLEntityExtractor.parseEntitiesFromSparseUntokenizedXML(titleText, PROTEIN_TAG,
470                        PROTEIN_TAG, pmid + "-t", DOCUMENT_COLLECTION_ID, ClassMentionTypes.PROTEIN, annotator, annotationSet);
471    
472                nextTitleDocument.setAnnotations(titleAnnotations);
473    
474                List<TextAnnotation> abstractAnnotations = XMLEntityExtractor.parseEntitiesFromSparseUntokenizedXML(abstractText, PROTEIN_TAG,
475                        PROTEIN_TAG, pmid + "-a", DOCUMENT_COLLECTION_ID, ClassMentionTypes.PROTEIN, annotator, annotationSet);
476    
477                nextAbstractDocument.setAnnotations(abstractAnnotations);
478    
479                List<TextAnnotation> annotationsToReturn = new ArrayList<TextAnnotation>();
480                annotationsToReturn.addAll(titleAnnotations);
481                annotationsToReturn.addAll(abstractAnnotations);
482                return annotationsToReturn;
483    
484            } else {
485                System.err.println("ERROR -- YapexCorpusParser: PMIDS DO NOT MATCH!!");
486            }
487    
488            return null;
489        }
490    
491    //    /**
492    //     * args[0] = corpus file <br>
493    //     * args[1] = test or train <br>
494    //     * args[2] = connection properties file for annotation db
495    //     * 
496    //     * @param args
497    //     */
498    //    public static final void main(String[] args) {
499    //        boolean isTest = false;
500    //        if (args[1].equals("test")) {
501    //            isTest = true;
502    //        } else if (args[1].equals("train")) {
503    //            isTest = false;
504    //        } else {
505    //            System.err.println("ERROR -- YapexCorpusParser: invalid input parameter. Expecting test or train.");
506    //        }
507    //
508    //        try {
509    //            YapexCorpusParser ycp = new YapexCorpusParser(args[0], isTest, args[2]);
510    //        } catch (IOException e) {
511    //            e.printStackTrace();
512    //        }
513    //
514    //    }
515    
516        @Override
517        public void close() throws IOException {
518            corpusReader.close();
519        }
520    
521    }
```
